# P098: Cost of antimicrobial treatment in patients with bloodstream infection in an intensive care unit

**DOI:** 10.1186/2047-2994-2-S1-P98

**Published:** 2013-06-20

**Authors:** AO Paula, AC Oliveira, RF Rocha

**Affiliations:** 1 UFMG, Belo Horizonte, Brazil; 2 ENB, UFMG, Brazil; 3 Mater Dei Hospital, Belo Horizonte, Brazil

## Introduction

The study of costs associated with antimicrobials treatment of patients with bloodstream infection (BSI) are important to sustain implementation of preventive measures and increase patient safety.

## Objectives

Compare the direct cost of treatment of patients with BSI caused by methicillin-resistant and susceptible *Staphylococcus aureus* (MRSA and MSSA).

## Methods

A retrospective cohort study performed in an intensive care unit of a large hospital in Belo Horizonte. The population is comprised of patients with *Staphylococcus aureus* BSI from 2007 to 2011. Data were obtained through patients’ medical records, hospital infection control committee and hospital’s finance department. Therapy costs were grouped according to the type of treatment received: empirical (before the culture test result) or directed (according to the BSI causing agent). Descriptive and univariate analysis were performed. The hospital’s Ethics Committee approved the project.

## Results

62 patients were included, 31 in each group (MRSA and MSSA). For empirical treatment, due to the larger doses of meropenem used in MRSA patients at this stage, patients with MRSA had a statistically significant (p<0.005) larger cost ($1110.22) when compared to MSSA ones ($ 506.34). For directed and total treatment, difference between groups was not significant (directed treatment: MRSA $304.74 and MSSA $432.96; total treatment: MRSA $1061,01 and MSSA $829.40). This occurred due to the larger oxacillin dosages administered in patients with MSSA when compared with the treatment of vancomycin for patients with MRSA However, for both groups together, the costs of directed treatment ($327.57) were smaller than the costs of empirical treatment ($551.43) (p=0.000) showing that antimicrobial de-escalation reduced the directed costs.

## Conclusion

Bacterial resistance may influence the costs of antimicrobial treatment. The treatment targeting favored a rational use of antibiotics, reducing the costs after culture results.

## Disclosure of interest

None declared

